# Unveiling the proteome of the fasting heart: Insights into HIF-1 pathway regulation

**DOI:** 10.3389/fphys.2024.1462014

**Published:** 2024-10-14

**Authors:** Daniel Benak, Kristyna Holzerova, Frantisek Kolar, Miloslava Chalupova, Marketa Hlavackova

**Affiliations:** ^1^ Laboratory of Developmental Cardiology, Institute of Physiology of the Czech Academy of Sciences, Prague, Czechia; ^2^ Department of Physiology, Faculty of Science, Charles University, Prague, Czechia

**Keywords:** heart, fasting, proteome, HIF-1, PHD3

## Abstract

Fasting is a common dietary intervention known for its protective effects against metabolic and cardiovascular diseases. While its effects are mostly systemic, understanding tissue-specific changes in the heart is crucial for the identification of the mechanisms underlying fasting-induced cardioprotection. In this study, we performed a proteomic analysis of the fasting heart and attempted to clarify the molecular basis of fasting-induced cardioprotection. Our investigation identified a total of 4,652 proteins, with 127 exhibiting downregulation and 118 showing upregulation after fasting. Annotation analysis highlighted significant changes in processes such as lipid metabolism, the peroxisome pathway, and reactive oxygen species metabolism. Notably, the HIF-1 signaling pathway emerged as one of the focal points, with various HIF-1 targets exhibiting differential responses to fasting. Further experiments demonstrated downregulation of HIF-1α at both transcript and protein levels. Intriguingly, while gene expression of *Egln3* decreased, its protein product PHD3 remained unaffected by fasting. The unchanged levels of pro-inflammatory cytokines indicated that the observed reduction in *Hif1a* expression did not stem from a decrease in basal inflammation. These findings underscore the complex regulation of the well-established cardioprotective HIF-1 signaling within the heart during 3-day fasting.

## 1 Introduction

Ischemic heart disease is the leading cause of death worldwide, highlighting a critical area of concern in public health (WHO, 2020). Given this context, investigating the molecular basis of unconventional cardioprotective interventions, such as fasting, becomes increasingly relevant.

The 3-day fasting is an experimental cardioprotective model that protected rat hearts against major endpoints of acute ischemia-reperfusion injury. This intervention was also associated with increased ketogenesis represented by an elevated concentration of 3-hydroxybutyrate (BHB) and BHB/acetoacetate ratio in the myocardium (Snorek et al., 2012). Moreover, our previous experiments showed that transcripts of genes linked with cytoprotective pathways of ketone bodies exhibited altered RNA modifications in the heart post-fasting (Benak et al., 2024a), suggesting the involvement of epitranscriptomic regulation in fasting-induced cardioprotection (Benak et al., 2023). Remarkably, hypoxia-inducible factor 1α (HIF-1α), a renowned transcription factor known for its cardioprotective properties (Cai et al., 2008; Neckar et al., 2018), is also one of the transcripts bearing epitranscriptomic modifications (Shmakova et al., 2021), and conversely, it also influences the expression of numerous epitranscriptomic regulators (Thalhammer et al., 2011).

The protective role of HIF-1α has been described in many cardioprotective interventions, such as adaptation to chronic hypoxia (Alanova et al., 2024; Ostadal et al., 2021) or ischemic conditioning (Cai et al., 2008). However, the role of the HIF-1 pathway in fasting-induced cardioprotection is far from being understood.

Given this context, the objective of our study was to investigate the cardiac proteome of rats subjected to 3-day fasting to better understand the molecular basis of fasting-induced cardioprotection, with a focus on the regulation of the well-known cardioprotective HIF-1 pathway.

## 2 Materials and methods

### 2.1 Animals and experimental protocol

Adult (12-week-old) male Wistar rats were used in this study. All animals were housed in a controlled environment with a stable temperature (23°C) and a 12-h light-dark cycle (light from 6:00 a.m.) (Alanova et al., 2017). Rats in the experimental group were deprived of food for 3 days but had unrestricted access to water (Benak et al., 2024a). The control group had access to the standard chow diet *ad libitum*. The use of animals was approved and supervised by the Animal Care and Use Committee of the Institute of Physiology of the Czech Academy of Sciences (No. 66/2021).

### 2.2 Tissue processing

Rats were sacrificed by cervical dislocation. After swift excision of the hearts and washing in the cold (0°C) saline, hearts were dissected, and left ventricles (LV) were collected (Balkova et al., 2009). LV samples were weighted, frozen, and stored in liquid nitrogen until use.

### 2.3 Proteomic analysis

The proteomic analysis was performed by the Proteomics service laboratory as described previously (Benak et al., 2024a). Briefly, heart samples were pulverized in liquid nitrogen, solubilized in 1% SDS, and processed according to the SP4 no-glass bead protocol. About 500 ng of tryptic peptides were separated on a 50 cm C18 column using a 2.5 h elution gradient and were analyzed in data-independent acquisition (DIA) mode on an Orbitrap Exploris 480 (Thermo Fisher Scientific, United States) mass spectrometer equipped with a FAIMS unit. Raw files were processed in Spectronaut 14 (Biognosys, Switzerland) using the library created from data-dependent acquisition (DDA) runs of all samples and pooled sample fractionated to eight fractions by Pierce High pH Reversed-Phase Peptide Fractionation Kit (Thermo Fisher Scientific, United States). UniProt UP000002494_10116. fasta release 2021_01 proteome file was used.

Statistical comparison of protein expression was performed in fasting versus control groups in R environment (version 4.3.2.) using the package MSstats (version 4.10.0 (Kohler et al., 2023)). For estimation of variability between the tested groups, principal component analysis (PCA) was done with PCAtools R package. MSstats pipeline was run with settings recommended for DIA data analysis. Proteins with one expressed peptide were removed from the analysis prior to the linear model-based group comparison. Imputation of missing values was performed per peptide using accelerated failure time model. Imputation failed for peptides when a whole protein was missing in a run (Kohler et al., 2023). Median normalization approach and the Tukey’s polish method were used for the data normalization and summarization, respectively. Differentially expressed (DE) proteins fitting the soft cut-off of absolute log2 fold change (log2FC) 0.5 and the *p*-value less than 0.05 were selected for further analysis. Features with less than 3 replicates per group were excluded. An R package clusterProfiler (Wu et al., 2021) with over-representation analysis (ORA) and data from Gene Ontology (https://geneontology.org/), KEGG (www.kegg.jp), and Reactome (https://reactome.org/) databases were utilized for annotation of DE proteins.

Western blot analysis was used to verify proteomic findings by focusing on two key proteins involved in cardiac energy metabolism: pyruvate dehydrogenase kinase 4 (PDK4) and hexokinase 2 (HK2). PDK4 was selected due to its role in promoting fatty acid oxidation by inhibiting the pyruvate dehydrogenase complex, a metabolic shift typical during fasting. HK2, a key glycolytic enzyme, was chosen because its downregulation reflects reduced glucose utilization. Western blot results confirmed the upregulation of PDK4 and downregulation of HK2 in the hearts of fasting rats (Supplementary Figure S1), consistent with the results of the proteomic analysis.

### 2.4 RT-qPCR

RNA isolation, cDNA synthesis, and RT-qPCR were performed as described earlier (Holzerova et al., 2015). In short, the total RNA was extracted from each LV sample using RNAzol^®^ RT according to the manufacturer’s instructions. One μg of total RNA and random primers were used for cDNA synthesis with RevertAid H Minus First Strand cDNA Synthesis Kit (Thermo Fisher Scientific, United States) according to the manufacturer’s protocol. RT-qPCR was performed in 20 μL reaction volume on a LightCycler^®^ 480 (Roche Diagnostics, Switzerland) using TaqMan Gene Expression Assays (Thermo Fisher Scientific; listed in the supplements) and 5x HOT FIREPol Probe qPCR Mix Plus (NO ROX) (Solis Biodyne, Estonia) according to the manufacturer’s instructions with the following temperature profile: initial enzyme activation (15 min at 95°C) followed by 45 cycles of amplification (15 s at 95°C, 1 min at 60°C). For proper normalization of results (Benak et al., 2019), Tyrosin-3-monooxygenase/tryptophan 5 monooxygenase activation protein zeta (*Ywhaz*) and DNA topoisomerase I (*Top1*) were selected as suitable reference genes (Benak et al., 2024a).

### 2.5 Western blot

Tissue homogenization, protein separation, and immunodetection were performed as described previously with slight modifications (Pokorna et al., 2019). In short, protein loadings were 60 μg (HIF-1α), 20 μg (PHD3), 10 μg (PDK4, HK2). The membranes were incubated with primary and secondary antibodies: anti-HIF-1*α* (Novus Biologicals; NB100-479; 1:1,500; overnight), anti-PHD3 (Abcam; ab184714; 1:1,000; overnight), anti-PDK4 (Abcam; ab214938; 1:1000; overnight), anti-HK2 (Abcam; ab209847; 1:1000; overnight), and anti-rabbit (Bio-Rad; 170–6515; 1:10,000; 1 h). Chemiluminescence was measured by the ChemiDoc™ System (Bio-Rad, United States). Ponceau staining (Sigma-Aldrich; P7170) was used for normalization of Western blot data (Semenovykh et al., 2022), a method preferred over the use of housekeeping proteins as loading controls (Moritz, 2017).

### 2.6 Determination of proinflammatory cytokines

Enzyme-linked immunosorbent assay (ELISA) commercial kits were used to determine IL-6 (Invitrogen; BMS625) and TNFα (Invitrogen; BMS622) levels in LV homogenates (50 µL) according to the manufacturer’s instructions.

### 2.7 Statistics

The proteomic analysis included 5 biological replicates per group. Other experiments included 5–13 biological replicates per group. Statistical analyses were performed using GraphPad Prism 8 (GraphPad Software, Inc.). An unpaired two-sided Student’s t-test was used for the assessment of the statistical significance. The data were obtained from at least three experiments and are displayed as means ± standard deviation (SD). Results were recognized as statistically significant when the *p*-value reached < 0.05.

## 3 Results

**FIGURE 1:**
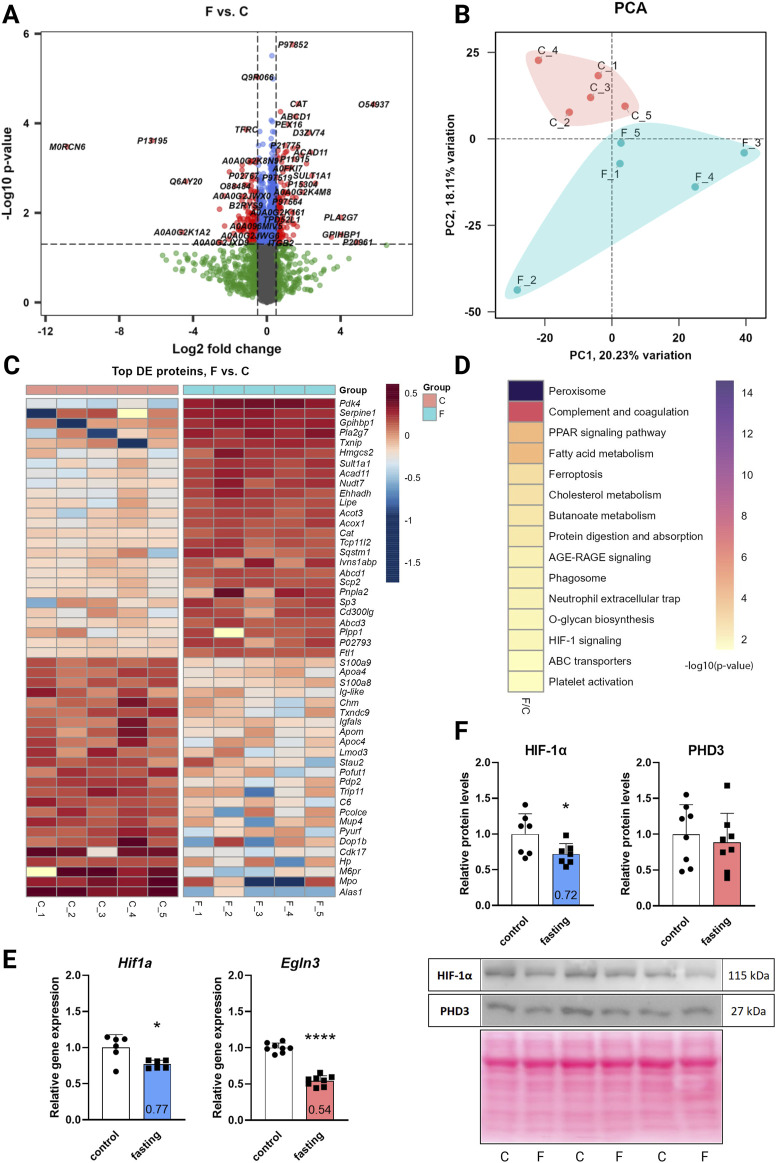
The effect of 3-day fasting on the proteome and HIF-1α and PHD3 (*Egln3*) levels. **(A)** Volcano plot of all 4,652 proteins detected by the proteomic analysis. **(B)** PCA (principal component analysis). **(C)** Heat map of the 50 top significantly changed proteins. **(D)** KEGG Annotation heat map of the main pathways affected by fasting **(E)** Gene expression levels of *Hif1a* and *Egln3* assessed by RT-qPCR. **(F)** Protein levels of HIF-1α and PHD3 assessed by Western blot and representative Western blot membranes. The average of the control values is set to 1. Values are means ± SD; n = 5–8; *p < 0.05, ****p < 0.0001 (t-test). C – control; F – fasting; *Egln3*/PHD3 – prolyl hydroxylase 3; HIF-1α – hypoxia-inducible factor 1α.

The DIA mass spectrometry analysis, providing higher sensitivity and protein coverage than the classic DDA, assessed the effect of 3-day fasting on the proteome in cardiac tissue. In total, 4,652 proteins were detected by proteomic analysis (low abundant proteins were masked by highly abundant contractile proteins). The total results of the DE analysis were visualized with a volcano plot (Figure 1A). The PCA (Figure 1B) showed a clear separation between fasting and control rat samples with 18% of explained variance, reflecting distinct proteomic profiles associated with the fasting state. A heatmap was prepared for the top 50 significant DE proteins selected by fold change (Figure 1C). Due to moderate variance between groups, DE proteins were selected according to soft cut-offs.

Out of the 4,652 proteins detected, 127 exhibited downregulation and 118 demonstrated upregulation after fasting. The initial annotation analysis of DE proteins revealed that the most striking changes occurred in lipid metabolism and peroxisome pathways (Table 1; Figure 1D), consistent with previous findings (Arumugam et al., 2023). These processes included up to 24 affected proteins (lipid metabolic process). Notably, also 5 proteins from the HIF-1 signaling pathway were altered: serpin family E member 1 (SERPINE1) and cyclin-dependent kinase inhibitor 1B (CDKN1B) were significantly upregulated in fasting hearts, while transferrin (TF), HK2, and transferrin receptor (TFRC) were significantly downregulated. Besides the HIF-1 signaling KEGG pathway, PDK4 and BCL2 interacting protein 3 (BNIP3), which are also targets of HIF-1, exhibited a significant increase in the hearts of fasting rats.

**TABLE 1:** Main pathways detected in relation to the differentially expressed proteins.

Database	ID	Pathway	Protein coding genes
GO	GO:0016042	lipid catabolic process	*Gpihbp1, Pla2g7, Acad11, Ehhadh, Lipe, Acox1, Abcd1, Scp2, Pnpla2, Abcd3, Hsd17b4, Apoa4, Acox3, Apoc1, Apoe, Acaa1a, Phyh, Hsd17b11, Ech1, Cyp27a1, Lonp2, Acot7, Mgll, Cbr1*
	GO:0005777	peroxisome	*Acad11, Ehhadh, Acox1, Cat, Abcd1, Scp2, Abcd3, Hsd17b4, Acox3, Tmem135, Acbd5, Pex3, Acaa1a, Pex14, Phyh, Pex12, Pxmp2, Pex11b, Ech1, Lonp2, Hmgcl*
	GO:0072593	ROS metabolic process	*Pdk4, Mpo, Hp, Acox1, Cat, Abcd1, Apoa4, Crp, Bnip3, Itgb2, App, Hk2, Cbr1*
	GO:0007568	aging	*Mpo, Hp, Cat, Scp2, Igfals, Hsd17b4, Krt16, Crp, Ctsl, Apoe, Ppp1r9a, Serpinf1, Itgb2*
Reactome	R-RNO-9609507	protein localization	*Abcd1, Pex16, Lonp2, Ehhadh, Pex12, Phyh, Ech1, Pex14, Acox1, Abcd3, Cat*
	R-RNO-9033241	peroxisomal protein import	*Lonp2, Ehhadh, Pex12, Phyh, Ech1, Pex14, Acox1, Cat*
	R-RNO-1474244	extracellular matrix organization	*P4ha2, Itgb2, Colgalt1, Plod3, Col3a1, Lum, Col1a1, Vtn, Prss1, Fgg*
	R-RNO-8978868	fatty acid metabolism	*Tecrl, Abcd1, Acad11, Ehhadh, Phyh, Acox1, Abcc1, Cyp4b1*
	R-RNO-72312	rRNA processing	*Rps29-ps16, Rps29-ps17, Rps29-ps15, Rpl22l1, Rpl18a, Rps29*
KEGG	rno04146	peroxisome	*Pex14, Abcd1, Pex12, Cat, Acox1, Ehhadh, Abcd3, Phyh, Ech1, Pex14, Scp2, Hsd17b4, Acox3, Pex3, Acaa1a, Hmgcl, Pxmp2, Pex11b*
	rno03320	PPAR signaling pathway	*Acox1, Ehhadh, Hmgcs2, Scp2, Acox3, Acaa1a, Cyp27a1*
	rno01212	fatty acid metabolism	*Acox1, Ehhadh, Scp2, Hsd17b, Acox3, Acaa1a*
	rno04066	HIF-1 signaling pathway	*Serpine1, Cdkn1b, Tf, Hk2, Tfrc*

While proteomic analysis suggested regulation of the HIF-1 signaling pathway, neither HIF-1α itself nor PHD3, the prolyl hydroxylase highly expressed in the heart, was captured by the proteomic analysis. This discrepancy arises primarily from methodological limitations rather than an intrinsic absence of expression. In cardiac tissue, the detection of low-abundance proteins such as HIF-1α can be masked by the presence of highly abundant contractile proteins. To address this, we investigated the gene expression and protein levels of HIF-1α and its degrading enzyme PHD3 (*Egln3*) using RT-qPCR (Figure 1E) and Western blot (Figure 1F). At the gene expression level, both transcripts were downregulated – *Hif1a* by 23% and *Egln3* by 46%. However, at the protein level, only HIF-1α was affected by 3-day fasting (28% decrease), while PHD3 remained stable.

Western blot experiments for verification of proteomic data confirmed upregulation of PDK4 by 571% and downregulation of HK2 by 16% (Supplementary Figure S1).

Additional RT-qPCR experiments revealed the gene expression profiles of the following HIF-1 targets: *Adora2b*, *Gapdh*, *Nos2*, *Nox4*, and *Vegfa* were not significantly affected by fasting; *Pdk4* and *Hmox1* were upregulated by 722% and 138%, respectively; and *Hk2* with *Gja1* were downregulated by 63% and 16%, respectively (Supplementary Figure S2).

Considering the observed decrease in *Hif1a* expression and the fact that: a) inflammation typically upregulates *Hif1a* expression (Palazon et al., 2014), and b) fasting correlates with a decrease in the basal levels of circulating pro-inflammatory cytokines (Aksungar et al., 2007), we subsequently examined the protein levels of the pro-inflammatory cytokines interleukin-6 (IL-6) and tumor necrosis factor-alpha (TNFα) in the myocardium. However, analysis revealed no significant alterations in their levels (Supplementary Figure S3).

## 4 Discussion

The 3-day fasting is an established experimental model inducing cardioprotection in rats (Snorek et al., 2012). However, given the elevated metabolic rate and other physiological differences in rats compared to humans (such as protein turnover rate or relative life expectancy), drawing direct comparison between fasting durations in the two species is challenging. Nevertheless, this model can be roughly analogized to an extended multi-week fast in humans (Wilhelmi de Toledo et al., 2019). The severity of this model limits the relevance of our findings for human physiology. However, the molecular mechanisms underlying fasting-induced cardioprotection could reveal potential therapeutic targets and lead to the development of novel clinical strategies (such as new pharmacological interventions) relevant to human health.

**FIGURE 2:**
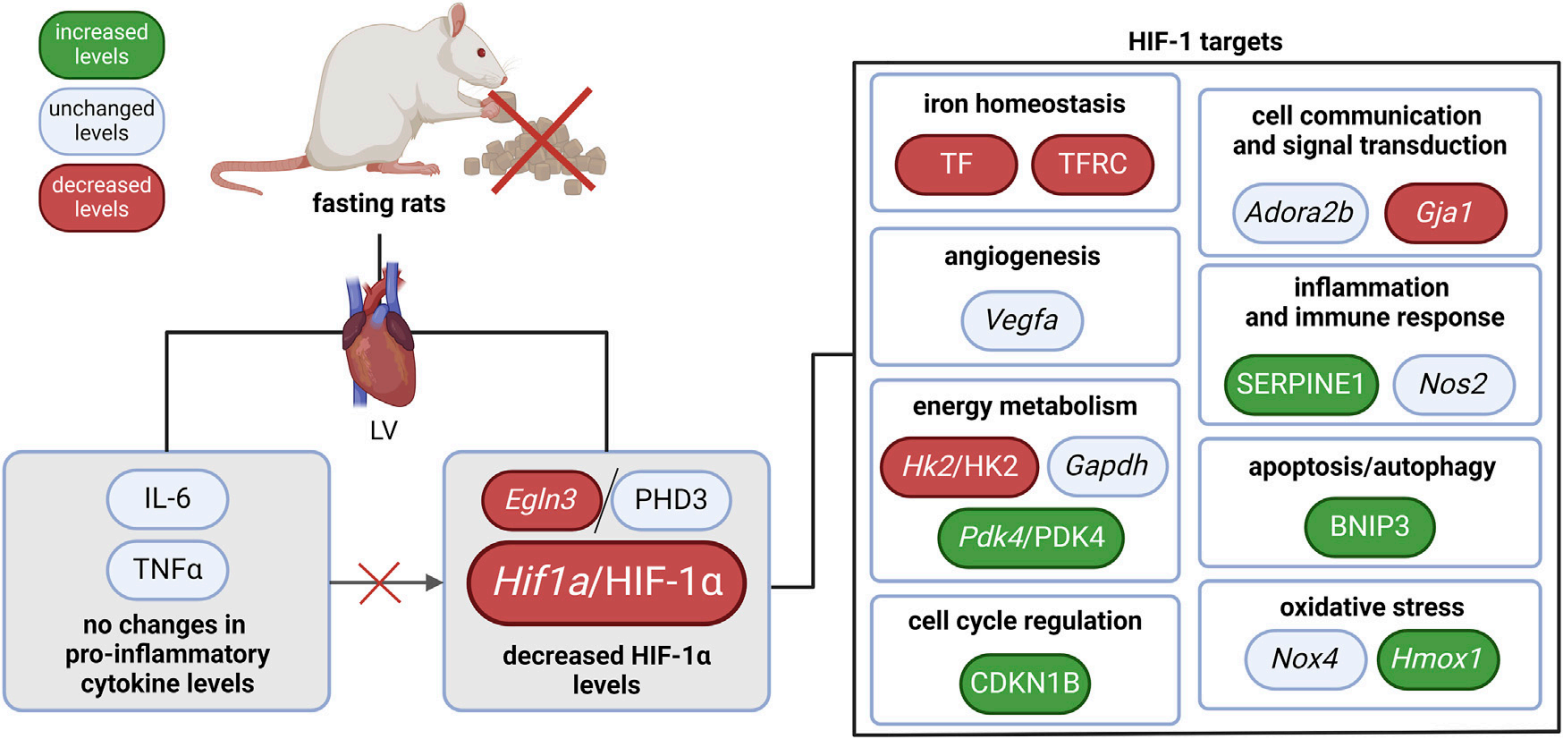
The effect of 3-day fasting on the HIF-1 signaling in the heart. *Adora2b*–adenosine A2b receptor; BNIP3 – BCL2 interacting protein 3; *Egln3*/PHD3 – prolyl hydroxylase 3; *Gapdh* – glyceraldehyde-3-phosphate dehydrogenase; *Gja1* – gap junction protein alpha 1; *Hif1a*/HIF-1α – hypoxia-inducible factor 1α; *Hk2*/HK2 – hexokinase 2; *Hmox1* – heme oxygenase 1; IL-6 – interleukin-6; LV – left ventricle; *Nos2* – nitric oxide synthase 2; *Nox4* – NADPH oxidase 4; *Pdk4* – pyruvate dehydrogenase kinase 4; TNFα – tumor necrosis factor α; *Vegfa* – vascular endothelial growth factor A.

In our study, we revealed that the HIF-1 signaling pathway was affected by 3-day fasting. HIF-1α was reduced on both mRNA and protein level. Moreover, several HIF-1 targets were regulated as well (Figure 2): TF with TFRC (iron homeostasis), *Hk2*/HK2 (energy metabolism), and *Gja1* (cell communication and signal transduction) were decreased, while *Pdk4*/PDK4 (energy metabolism), SERPINE1 (also known as PAI-1; inflammation and immune response), CDKN1B (also known as p27; cell cycle regulation), BNIP3 (apoptosis/autophagy), and *Hmox1* (oxidative stress) were increased. These data show that the HIF-1 signaling in fasting hearts differ from the classical protective HIF-1 signaling in hypoxic tissues (Kegg pathway, 2021).

Our findings align with those of Arumugam et al. (2023) who also reported the regulation of hypoxic signaling in fasting mice. Their study investigated the effects of three long-term (6-month) intermittent fasting protocols: 12-h daily fast, 16-h daily fast, and alternate-day fasting. Among the 503 DE proteins in heart tissues across all three dietary regimens, only 60 proteins consistently overlapped. These results underscore the high sensitivity of the cardiac proteome to the duration and type of fasting regimen. Hence, the relatively low overlap with our results is not surprising. Specifically, out of the proteins detected in the HIF-1 signaling pathway after 3-day fasting, only HK2 overlapped between the two studies. This protein was significantly decreased in all fasting interventions. Notably, the involvement of HK2 in cardioprotection has been demonstrated (Halestrap et al., 2015).

Conflicting data regarding the relationship between fasting or ketone bodies and HIF-1α are present in the literature. Fasting, ketogenic diet, or administration of BHB was primarily associated with increased HIF-1α levels due to the accumulation of succinate, a known inhibitor of prolyl hydroxylases (Puchowicz et al., 2008). Yet, treatment of mouse primary cardiomyocytes with high doses of BHB significantly decreased HIF-1α levels under hypoxic (1% O_2_, 12 h) conditions (Ma et al., 2022). Another study showed that HIF-1α regulation might vary between different tissues depending on fasting duration. Northern elephant seal pups (*Mirounga angustirostris*) exhibited increased *Hif1a* mRNA levels in adipose tissue after prolonged fasting (3, 5, and 7 weeks), whereas in muscle tissue, *Hif1a* levels were unaffected after a 3-week fast, decreased after a 5-week fast, and increased after a 7-week fast (Sonanez-Organis et al., 2013). In our experiments, we found downregulation of HIF-1α on both transcript and protein levels.

Regarding PHD3/*Egln3*, no changes in its gene expression were reported after 16-h fasting in mouse hearts (Yoon et al., 2020), which contrasts with our observation of decreased cardiac levels of *Egln3* after the 3-day fast. However, we also did not observe any alterations at the protein level. Thus, HIF-1α regulation in the fasting heart seems to be PHD3-independent. Moreover, the expression of *Hif1a* was altered already at the transcriptional stage. Since inflammation is known to positively affect *Hif1a* expression (Gorlach, 2009), we hypothesized that the decrease in *Hif1a* mRNA levels could be triggered by fasting-induced decrease in basal inflammation, which has been associated with fasting (Aksungar et al., 2007). However, stable myocardial levels of pro-inflammatory cytokines IL-6 and TNFα did not support this hypothesis. Thus, the reduction in *Hif1a* mRNA levels is likely driven by other molecular mechanisms, such as the action of other transcription factors, epigenetic or epitranscriptomic modifications, regulation by non-coding RNAs, etc. Notably, the emerging field of epitranscriptomics, which explores RNA modifications that modulate gene expression beyond the classical framework of the central dogma, presents a promising avenue for future research (Benak et al., 2024b). Several studies have already highlighted the reciprocal regulatory interactions between N^6^-methyladenosine (m^6^A) modification and HIF-1 (Shmakova et al., 2021; Thalhammer et al., 2011; Li et al., 2021; An et al., 2023; Liang et al., 2022; Nan et al., 2023), underscoring the potential significance of this mechanism in HIF-1 regulation during fasting.

Overall, reduced HIF-1α levels were not accompanied by a uniform downregulation of HIF-1 targets, suggesting a complex interaction between HIF-1 signaling and other molecular pathways during fasting. Thus, the precise role of HIF-1 regulation in fasting-induced cardioprotection, its dynamics at specific stages of fasting, and its interactions with other protective pathways remain key areas for further exploration.

The consensus suggests that fasting confers cardioprotective benefits. However, available data has highlighted a fine balance between its positive and detrimental effects. For instance, a 3-day fasting period exhibited cardioprotective effects in rats (Snorek et al., 2012), whereas an 18-h fasting window was found to be detrimental to rat heart health resulting in larger infarct size (Liepinsh et al., 2014). Taken together, these conflicting results highlight the need to investigate the interaction of fasting and HIF-1 signaling in the heart. Its elucidation could help us understand the complex molecular background of cardioprotection and reduce the morbidity and mortality of ischemic heart disease.

## 5 Conclusion

In this study, we explored the intricate dynamics of the fasting heart’s proteome, particularly focusing on the regulation of the HIF-1 pathway. Our proteomic analysis revealed significant alterations in 245 proteins, underscoring the profound impact of a 3-day fasting regimen on cardiac protein expression. Among these, the modulation of the HIF-1 signaling pathway, a known mediator of cardioprotection, was notably significant. This modulation was characterized by a downregulation of HIF-1α at both transcript and protein levels, suggesting a nuanced regulatory mechanism in response to fasting. Furthermore, our findings on the unchanged protein levels of PHD3, despite the decreased gene expression of its encoding gene *Egln3*, added complexity to our understanding of HIF-1α regulation during fasting. Stable levels of pro-inflammatory cytokines suggested that the reduction in *Hif1a* expression is not due to a decrease in basal inflammation. Moreover, the HIF-1 targets were distinctly regulated, suggesting a complex interplay between HIF-1 signaling and other molecular pathways during fasting. These observations provide insights into the tissue-specific adaptations of the heart to fasting and highlight the potential of the HIF-1 signaling in this cardioprotective intervention.

## Data Availability

All relevant data, associated protocols, and materials are within the manuscript or at Webshare: https://webshare.cz/#/group/lZ9ZyCAUaZ/. The mass spectrometry proteomics data have been deposited to the ProteomeXchange Consortium via the PRIDE partner repository with the dataset identifier PXD050458. If any additional information is needed, it will be available upon request from the corresponding author.
