# Supplementary material for: Unveiling the proteome of the fasting heart: Insights into HIF-1 pathway regulation
Source: Front Physiol. 2024 Oct 14;15:1462014. doi: 10.3389/fphys.2024.1462014 (PMC11513464; doi:10.3389/fphys.2024.1462014)
Supplement: Supplementary file 1 [file DataSheet1.PDF]

Supplementary materials

Materials and Methods

2.4. RT-qPCR

The following TaqMan gene expression assays were used in this manuscript: *Adora2b* (adenosine A2b receptor, 4331182, Rn00567697_m1); *Egln3* (prolyl hydroxylase 3, 4331182, Rn00571341_m1); *Gapdh* (glyceraldehyde-3-phosphate dehydrogenase, 4331182, Rn01775763_g1); *Gja1* (gap junction protein alpha 1, 4351372, Rn06415115_s1); *Hif1a* (hypoxia-inducible factor 1 alpha, 4331182, Rn01472831_m1); *Hk2* (hexokinase 2, 4331182, Rn00562457_m1); *Hmox1* (heme oxygenase 1, 4351372, Rn02117416_s1); *Nos2* (nitric oxide synthase 2, 4331182, Rn00561646_m1); *Nox4* (NADPH oxidase 4, 4331182, Rn00585380_m1); *Pdk4* (pyruvate dehydrogenase kinase 4, 4331182, Rn00585577_m1); *Top1* (DNA topoisomerase, 4331182, Rn00575128_m1); *Vegfa* (vascular endothelial growth factor A, 4331182, Rn01511602_m1); *Ywhaz* (tyrosin-3-monooxygenase/tryptophan 5 monooxygenase activation protein zeta, 4448484, Rn00755072_m1).

Figures

Figure S1.

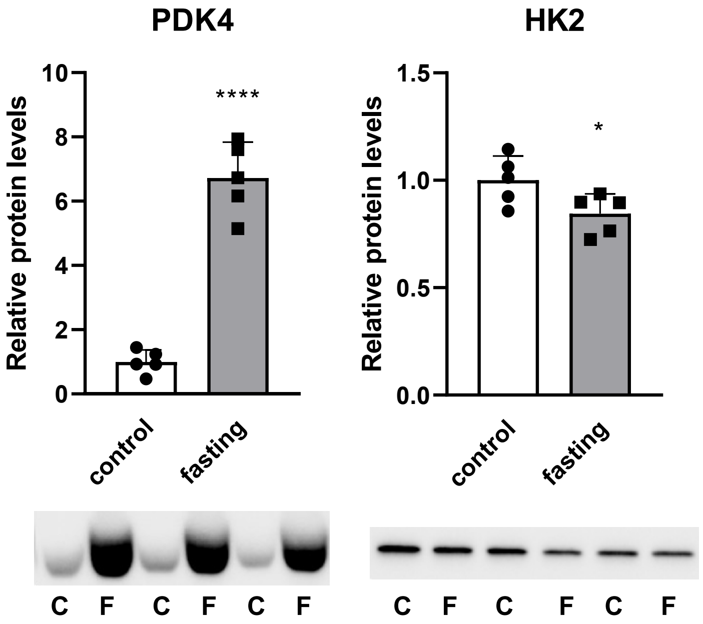

Figure S2.

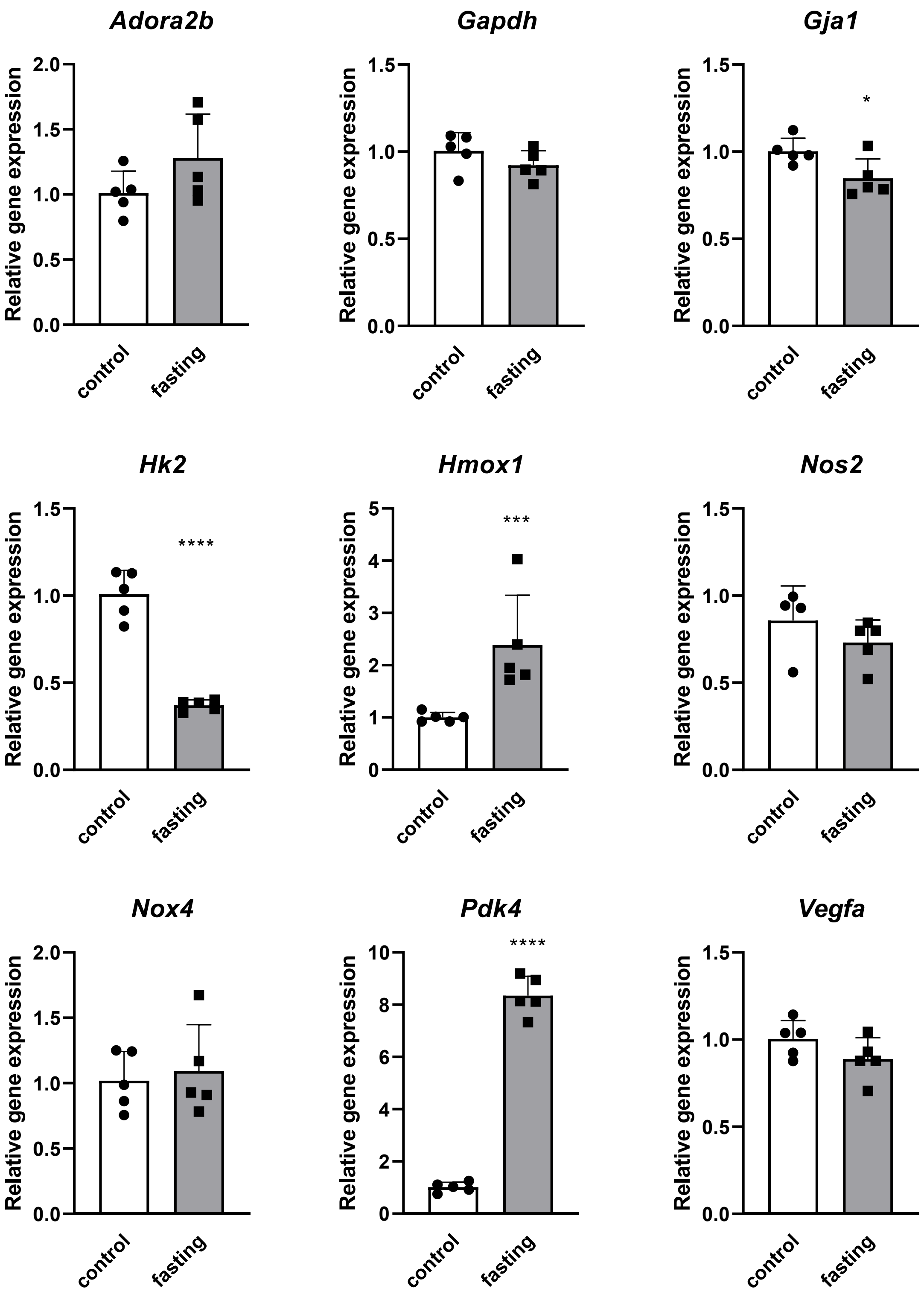

Adora2b
Gapdh
Gja1
Hk2
Hmox1
Nos2
Nox4
Pdk4
Vegfa
Relative gene expression
control
fasting
*
****
***
****

Figure S3.

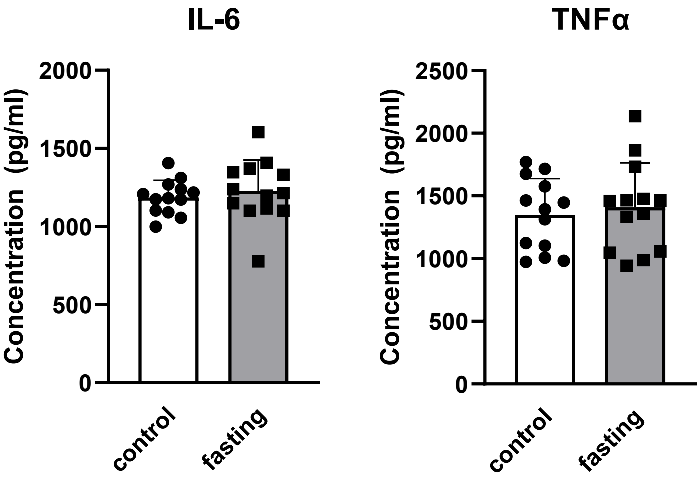

IL-6
TNFα
Concentration (pg/ml)
2000
1500
1000
500
0
2500
control
fasting

Figure captions

Fig. S1.: Effect of 3-day fasting on protein levels of expressions of HIF-1 targets in the left ventricle with representative Western blot membranes – verification of proteomic data. Values are means ± SD; n = 5; * $p < 0.05$; **** $p < 0.0001$ (t-test). HK2 – hexokinase 2; PDK4 – pyruvate dehydrogenase kinase 4.

Fig. S2.: Effect of 3-day fasting on gene expressions of HIF-1 targets in the left ventricle. Values are means ± SD; n = 5; * $p < 0.05$; *** $p < 0.001$; **** $p < 0.0001$ (t-test). *Adora2b* – adenosine A2b receptor; *Gapdh* – glyceraldehyde-3-phosphate dehydrogenase; *Gja1* – gap junction protein alpha 1; *Hk2* – hexokinase 2; *Hmox1* – heme oxygenase 1; *Nos2* – nitric oxide synthase 2; *Nox4* – NADPH oxidase 4; *Pdk4* – pyruvate dehydrogenase kinase 4; *Vegfa* – vascular endothelial growth factor A.

Fig. S3.: Effect of 3-day fasting on pro-inflammatory cytokines in the left ventricle. Values are means ± SD; n = 13. IL-6 – interleukin-6; TNFα – tumor necrosis factor alpha.
